# An integrated approach of field, weather, and satellite data for monitoring maize phenology

**DOI:** 10.1038/s41598-021-95253-7

**Published:** 2021-08-03

**Authors:** Luciana Nieto, Raí Schwalbert, P. V. Vara Prasad, Bradley J. S. C. Olson, Ignacio A. Ciampitti

**Affiliations:** 1 Department of Agronomy, 2004 Throckmorton Plant Science Center, Kansas State University, 1712 Claflin Road, Manhattan, KS 66506 USA; 2 Sustainable Intensification Innovation Lab, Kansas State University, 108 Waters Hall, 1603 Old Claflin Place, Manhattan, KS 66506 USA; 3 Department of Biology, Kansas State University, Chalmers Hall, 1711 Claflin Road, Manhattan, KS 66506 USA

**Keywords:** Data processing, Machine learning

## Abstract

Efficient, more accurate reporting of maize (*Zea mays* L.) phenology, crop condition, and progress is crucial for agronomists and policy makers. Integration of satellite imagery with machine learning models has shown great potential to improve crop classification and facilitate in-season phenological reports. However, crop phenology classification precision must be substantially improved to transform data into actionable management decisions for farmers and agronomists. An integrated approach utilizing ground truth field data for maize crop phenology (2013–2018 seasons), satellite imagery (Landsat 8), and weather data was explored with the following objectives: (i) model training and validation—identify the best combination of spectral bands, vegetation indices (VIs), weather parameters, geolocation, and ground truth data, resulting in a model with the highest accuracy across years at each season segment (step one) and (ii) model testing—post-selection model performance evaluation for each phenology class with unseen data (hold-out cross-validation) (step two). The best model performance for classifying maize phenology was documented when VIs (NDVI, EVI, GCVI, NDWI, GVMI) and vapor pressure deficit (VPD) were used as input variables. This study supports the integration of field ground truth, satellite imagery, and weather data to classify maize crop phenology, thereby facilitating foundational decision making and agricultural interventions for the different members of the agricultural chain.

## Introduction

Maize (*Zea mays* L.) is one of the leading grain crops, with 193 M ha harvested globally and more than 33 M ha in the United States (US) during the 2018 growing season. US maize production is largely concentrated in the central region, accounting for roughly 85% of total US production^1^. The US Department of Agriculture (USDA) via the National Agricultural Statistics Service (NASS) releases a weekly report for in-season crop progress, termed Crop Progress and Report Conditions (CPRC), which provides a subjective estimate of crop phenology and condition in major US producing states^1^. Estimates from the CPRC are based on survey data gathered on a weekly basis from an extensive network of regional agricultural agents based on their field observations^2^. Although the data is informative, the collection process is labor intensive, time consuming, financially ineffective, and subject to bias^3,4^. With challenges eminent due to funding restrictions and travel limitations, new approaches should be pursued, tested, and quickly implemented.

An extensive record of scientific literature portrays the relevance of implementing well-timed management practices to improve yields and input use efficiency, such as timely irrigation, fertilization, crop protection, and harvest^5–7^. Remotely sensed satellite data presents a significant opportunity to improve timely agricultural interventions and monitoring of crop vegetation. With various spectral, radiometric, temporal and spatial resolutions, satellites function as a critical source of data to aid tracking of field crop phenology progress^8–10^. Combining satellite data features permit the generation of different vegetation indices (VIs), such as the Normalized Difference Vegetation Index (NDVI)^11^, Enhanced Vegetation Index (EVI)^12^, Green Chlorophyll Vegetation Index (GCVI)^13^, Global Vegetation Moisture Index (GVMI)^14^ and Normalized Difference Water Index (NDWI)^15^, among many others. These VIs describe changes in vegetation dynamics and are correlated with plant traits such as leaf area index, leaf chlorophyll concentration, and canopy water content^13–18^. Integrating remote sensing with ground truth and weather data has great potential to advance science and improve overall prediction of crop yield, in-season progress, and crop phenology^2,19,20^.

The development of programs such as Google Earth Engine^21^ facilitates dataset manipulation and analysis by integrating all assets in one place. Directly working in the cloud without downloading large sets of data and using a parallel processing approach allows computation across a large number of machines^22^. This new tool accelerates analysis of remote sensing projects over large areas and facilitates real-time crop behavior and progress exploration^21^. Of the many tools used to perform classifications, the random forest (RF) algorithm^23^ presents steady performance with large^24^ and unbalanced datasets^25^ in addition to modeling non-linearity correlations among the feature space and the dependent variable.

Recent studies focused on predicting field crop phenology, particularly in maize and other field crops^2,26^, concentrated on providing estimates for dormancy, green-up, mid-season growth and development, day of senescence, and end of the season. Although these metrics are convenient from a remote sensing analytical viewpoint, more detailed phenology descriptions^27,28^ are typically desirable to provide timely actionable agricultural decisions and interventions. Due to field data availability, this study is focused on the Southwest (SW) Agricultural District in Kansas, US. Ground truth data was provided by an industry partner, comprising a large dataset on crop phenology for maize fields during the 2013–2018 growing seasons. Therefore, the aim of this research study was to evaluate a classification of satellite-derived maize crop phenology and integrate in-season weather information to develop a classification model benchmarked with field survey data. To achieve this overarching goal, we established the following objectives: (i) model training and validation—understand how different variables affect the model performance and identify the best combination of spectral features, weather parameters, geolocation and ground truth data, resulting in a model with the highest accuracy across years at each season segment (step one); and (ii) model testing—post-selection model performance evaluation for (a) each phenology class with unseen data (hold-out cross-validation); (b) temporal transferability; (c) spatial transferability (step two).

## Materials and methods

### Study area and environmental conditions

**Figure 1:**
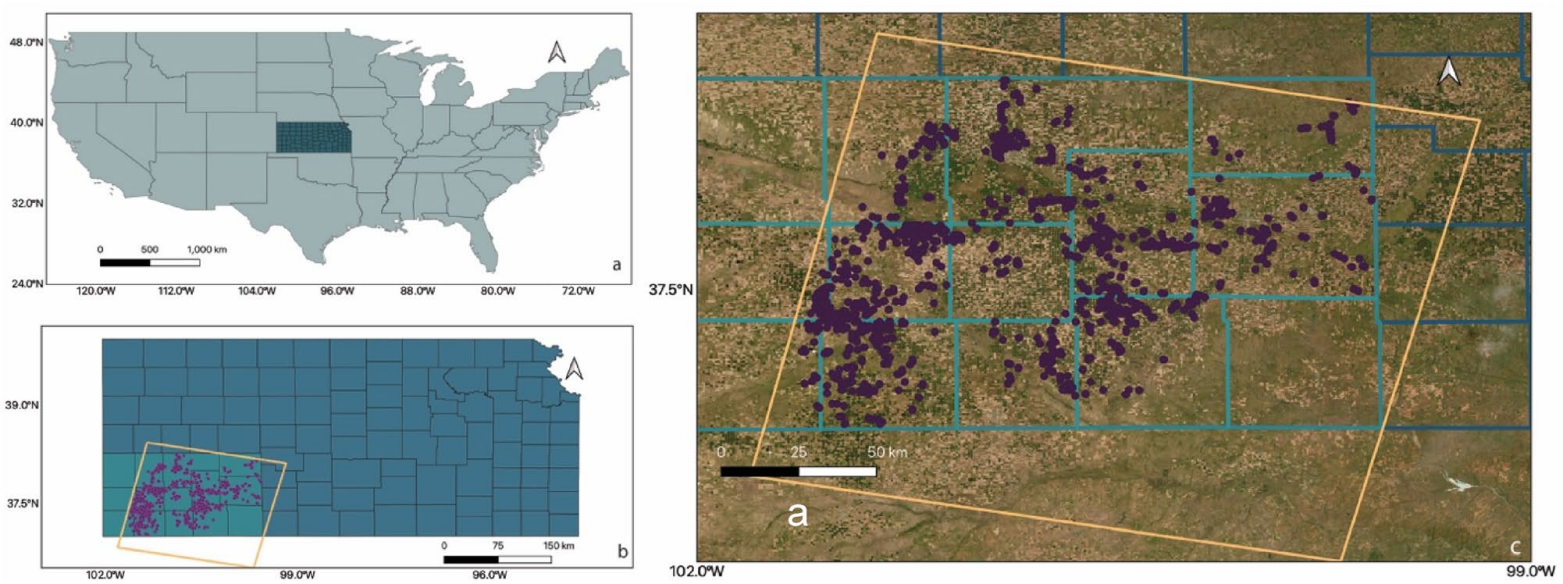
(**a**) area of study highlighting, the USA, in green and Kansas in blue (**b**) Area of study. The purple points correspond to phenology measurements in Kansas's Southwest Agricultural District; the orange area corresponds to Landsat 8 tile 30/34, which covers the area under study. (**c**) Zoomed-in highlight of the Southwest Agricultural District (light blue area) and the location of each phenology measurement. Maps were created using ArcGIS software version 10.7 by Esri. ArcGIS and ArcMap are the intellectual property of Esri and are used herein under license. Copyright Esri. All rights reserved. For more information about Esri software, please visit www.esri.com.

This research was conducted in the SW Agricultural District, comprising a total of 10 counties, Kansas, US. Annual normal precipitation in this area ranges from 381 to 635 mm from west to east, with the southwest corner (bordering the states of Oklahoma and Colorado) presenting the lowest precipitation (Fig. 1). Average mean daily temperature fluctuates from 12 to 14 °C, with the average minimum ranging from 7.5 to 15 °C and the average maximum between 19 and 22 °C^29^. The maize growing season spans from late April (sowing) to late October (harvest), with yields usually enhanced by groundwater irrigation from the Ogallala Aquifer^30^, which currently has more than half million of hectares under irrigation^31^.

### Data gathering and feature engineering

#### Surface reflectance data

Surface reflectance data was accessed via Google Earth Engine (GEE). Landsat 8, 30 m spatial resolution, Surface Reflectance (SR) collection, tier 1, was retrieved from the GEE data repository.

Images from 2013 to 2018 from May to September (season segment, May = 1, June = 2, July = 3, August = 4, September = 5) each year were collected for the path/row 30/34. Starting the season in May and extending it until September were supported by the Start of Season Time (SOST) and End of Season Time (EOST) analyses of the NDVI each year^32^. Images with ≤ 30% land cloud cover were selected, and remaining clouds and shadows were masked by taking advantage of the pixel quality band in the SR product. Five indices were calculated (NDVI, EVI, GCVI, NDWI and GVMI) over each of these images, and the mean value per index at each field (“Field data collection and feature engineering”) was stored by season segment.

#### Meteorological data

Gridded surface meteorological data from GEE archives was used to extract weather variables^33^. This dataset integrates high-resolution spatial data (2.5 arc minute, ~ 4 km) from PRISM^34^ with daily temporal resolution data from the North American Land Data Assimilation System (NLDAS). The main weather variables gathered were precipitation, mean minimum and maximum temperature, and vapor pressure deficit (VPD). The Growing Degree Units (GDU) were calculated using the following equation: GDU = [(Max. temperature (C) − Min. temperature (C))/2] − 10 °C (Base temperature)^35^.

#### Field data collection and feature engineering

The initial dataset provided by Crop Quest Inc. included more than 70,000 fields distributed across the states of Kansas, Oklahoma, Colorado, and Nebraska (USA). The first step was to select only the maize data points within the SW District in Kansas, presenting more than 20,000 fields (Fig. 1). This dataset contained geolocated maize phenology measurements (latitude and longitude), associated with the day of the year (DOY), for crop data collected from the 2013 to 2018 growing seasons. To not only avoid potential classifier issues due to the specificity level of the crop growth stages, but also provide informative data more readily translated into actionable decisions for potential agricultural interventions, the stages were re-grouped into nine classes (IV1, IV2, EV, LV, ER, LR1, LR2, LR3 and H), as presented in Table 1.

**Table 1:** Phenology classes (IV1-H) after re-grouping the phenological stages (VE–R6).

Class	Stages on the dataset
IV1	VE–V1
IV2	V2–V4
EV	V5–V8
LV	V9–Vnth
ER	VT–R3
LR1	R4
LR2	R5
LR3	R6
H	Harvest

*IV1* initial vegetative 1, *IV2* initial vegetative 2, *EV* early vegetative, *LV* late vegetative, *ER* early reproductive, *LR1* late reproductive 1, *LR2* late reproductive 2, *LR3* late reproductive 3, *H* harvest.

Remaining data points were geographically aligned and merged with polygon shapefiles containing the field boundaries. This new file was uploaded as an asset into GEE and used to summarize the satellite and weather information within each field. Additionally, as an approximation, a filter was applied to separate the center-pivot irrigation fields from rainfed agricultural farms. This filter was built using the Canny Edge Detector^36^. Although this technique returned nearly all fields under irrigation, some omission errors were present due to the different types of irrigation systems; only circular pivots of 500 m radius were extracted.

The final dataset included 21 independent variables and Growth Class as the dependent variable used to train and validate the models (full description in Supplementary Appendix A, Supplementary Table A).

### Model training and validation, random forest classification model

Random forest (RF) has been extensively used in the remote sensing field to solve classification problems. This method has been proven to be easy to train with a little or non-computational cost while also dealing with complex interactions and highly correlated variables^37,38^. Furthermore, RF is extensively documented in the scientific literature to outperform other methods. In the research conducted by Ref.^39^, testing 178 classifiers, RF resulted in the classifier with the best performance. Analysis conducted by Refs.^37,40,41^ among others, presented the same outcome.

Following the abovementioned rationale, all models were trained using a RF classification algorithm from *scikit learn* software^42^ in a Python 3.8.0 environment^43^. This algorithm was adjusted by fine-tuning hyperparameters, executing a grid search to find the best data combinations, once the best features were obtained (more details about feature selection can be find later on “Effects of feature selection on model performance”) The main hyperparameters explored in our analysis were the following: bootstrap, maximum depth of the tree, maximum number of features for the best split, minimum samples per leaf, minimum samples to perform the split, and the number of estimators^23^. The best model considered the following: bootstrap = true, maximum depth of the tree = 50, maximum number of features for the best split = 9, minimum samples per leaf = 2, minimum samples to perform the split = 4, and the number of estimators = 50.

To evaluate the classifier, datasets were split into training, validation, and test sections. The proportion retained for training and validation was 80% of the total (of this 80%, 80% were used as training and 20% as validation), and 20% was used as a test for model evaluation, the numbers for each class each year can be find in Supplementary Appendix C, Supplementary Table A. An example of the geographical distribution for field observations is presented as supplementary material (Supplementary Appendix B, Fig. 1). The selected proportions resulted from a sensitivity analysis using the entire dataset spanning from 2013 to 2018 period with all variables (later referred to as the Full model). In this analysis, the overall accuracy was used as a metric to measure model performance, with data proportions adjusted at increments of 10% (from 10 to 90%, to find the optimal combination for training and validation (80/20%).

### Model testing

#### Performance measurements

For this study, model classification performance was evaluated using five different metrics calculated using the test portion of the dataset. These metrics were as follows: precision, the number of true positives divided by the sum of true and false positives; recall, the number of true positives divided by the sum of true positives and false negatives; overall accuracy metric, the sum of all correctly classified elements divided by total elements; kappa coefficient of agreement, a measure describing how well the model is performing compared to the randomness (taking into account the possibility of agreement occurring by chance); and finally, F-score, the harmonic mean of the precision and recall^44^. The harmonic mean is biased towards observations with lower values. When all values have the same magnitude, the harmonic and arithmetic means are equivalent. The F-score has a maximum value of 1, representing perfect precision. Therefore, the F-score is maximized by simultaneously maximizing precision and recall.

The out of bag score (OOB) was computed in addition to evaluating the performance of the model. This measure computes the average misclassification ratio of non-training samples. The importance of this metric relies on offering an unbiased assessment of classification performance, with values closer to 1 indicating good performance^37^.

#### Effects of feature selection on model performance

The openly exploration of different combinations of variables to address model performance was motivated by two main reasons. First to understand how different features affect the performance of the model^45^; second to overcome any potential bias resulting from a feature importance analysis. This technique has been proven to favor variables presenting high cardinality^46,47^, condition present on the dataset for geographical features as well as growth DOY. Nonetheless a feature importance was calculated for one of the models to observe the behavior.

To test this abovementioned point, the following variable combinations were used for each year and for each season segment: all bands (B) including spectral bands 2, 3, 4, 5, 6, 7, 10, and 11 for Landsat 8; all bands and all weather parameters (BW), including the mentioned spectral bands, precipitation, maximum and minimum temperature, VPD, and GDU; all indices and all bands (VIB), including the mentioned spectral bands and NDVI, EVI, GCVI, GVMI, and NDWI as vegetation indices; all indices and weather parameters (VIW), including the five above mentioned weather parameters and five vegetation indices; only weather parameters (W), testing five weather parameters; only indices (VI), including only five vegetation indices; indices, weather parameters, and bands together (FULL), and finally a model only containing latitude, longitude and day of the year (LLD). The models most stable across years and within season (VIW; W; VI) were trained again analyzing all possible interactions between the variables presented. Thus, we explored 31 combinations for the W models, 31 combinations for the VI models, and 1023 combinations for the VIW models. The number of models actually fitted was greater than the previous sum (31 + 31 + 1023) due to the grid search approach for fine-tuning the hyperparameters. In order to account for spatio-temporal correlation in the response variable induced by other features that may not be captured by remote sensing (surface reflectance) and weather data, all model combinations included field geolocation and DOY^48^.

Because not all crop phenology classes were present between season segments due to the characteristics of this variable, the accuracy values for each model during each season segment for each year were retrieved to better understand the performance within and between years.

#### Model performance under temporal and spatial transferability.

Following the completion of the first stage, the subsequent analyses were established to better understand the performance and stability of the final model. To learn about the stability of the model across years, the dataset was divided into training and validation based on year separation. For this evaluation, we grouped the 6 years (2013–2018) according to the weather trend combining average years (2014, 2017) with dry (2013, 2018) and wet ones (2015, 2016). The same metrics (Overall accuracy, OOB, precision, recall and F1-score) were utilized to test this analysis.

To explore the ability of the model while leading with spatial transferability^37^, the dataset was divided into training and validation based on spatial attributes, with a total of 40.519 elements (sum of all the phenological measurements across the 6 years). This resulted on two datasets, a larger one comprised by the fields located in the western part of the area under study (training) where fields are more aggregated. The validation dataset was smaller and composed by the fields in the east side of the geographic area.

## Results

### Best combination of features and model performance

**Figure 2:**
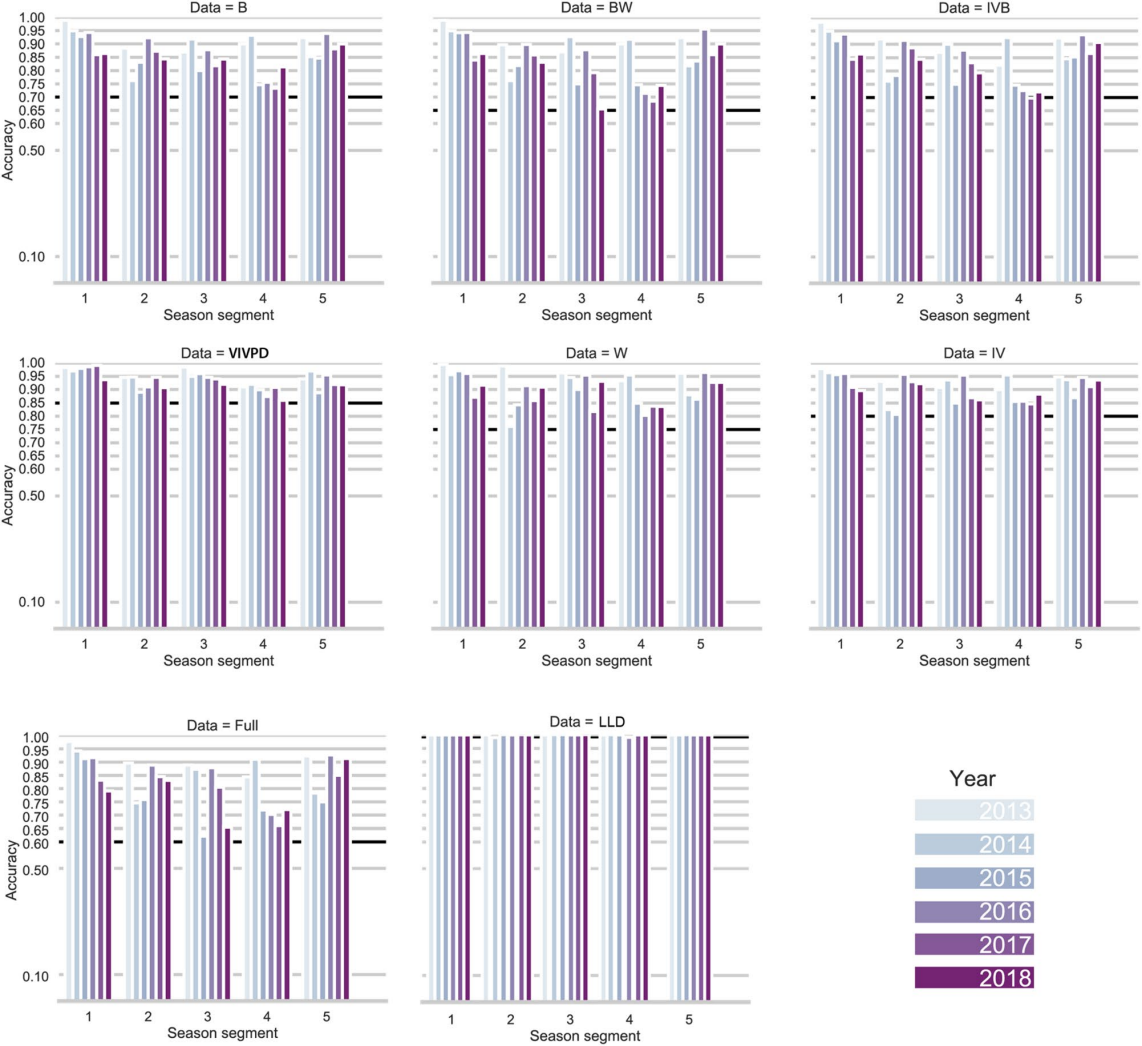
Accuracy values for each model during the season from 2013 to 2018. The black horizontal line shows the lowest value recorded. *B* spectral bands 2, 3, 4, 5, 6, 7, 10, and 11 for Landsat 8, *BW* spectral bands in addition to precipitation, maximum and minimum temperature, VPD, and GDU (weather parameters), *VIB* spectral bands in addition to NDVI, EVI, GCVI, GVMI, and NDWI (vegetation indices), *VIVPD* vegetation indices and vapor pressure deficit (VPD), *W* weather parameters, *VI* vegetation indices, *FULL* spectral bands, weather parameters, and vegetation indices, *LLD* latitude, longitude and doy.

All models explored in this study classified crop phenology classes with an accuracy above 70%, except for the model combining all 21 variables (accuracy below 60%) (Fig. 2). For this model, we also conducted a feature importance analysis (Supplementary Appendix B). Although most of the variables shared a similar importance, some were more relevant, such as latitude, longitude and growth doy, associated to each phenology measurement (variables with high cardinality), while others such as precipitation were deleterious. For remaining models, accuracy ranged from 70 to 100% within a season and between years. The models with the best performance between years and within season were (I) combination between vegetation indices and weather, (II) weather (III) vegetation indices, and (IV) latitude, longitude and doy. For these four models, all possible combinations between variables were tested. This resulted in two models with the best performances, one composed by 5 vegetation indices and only one weather parameter, VPD, where the accuracy assessment ranged from 86 to 98%; and the other model composed only by latitude, longitude and doy, with an accuracy of 100% for almost all the season segments—years (Fig. 2). Further discussion on this model is presented in in the discussion, later on this manuscript.

#### Classification using VIVPD model

**Figure 3:**
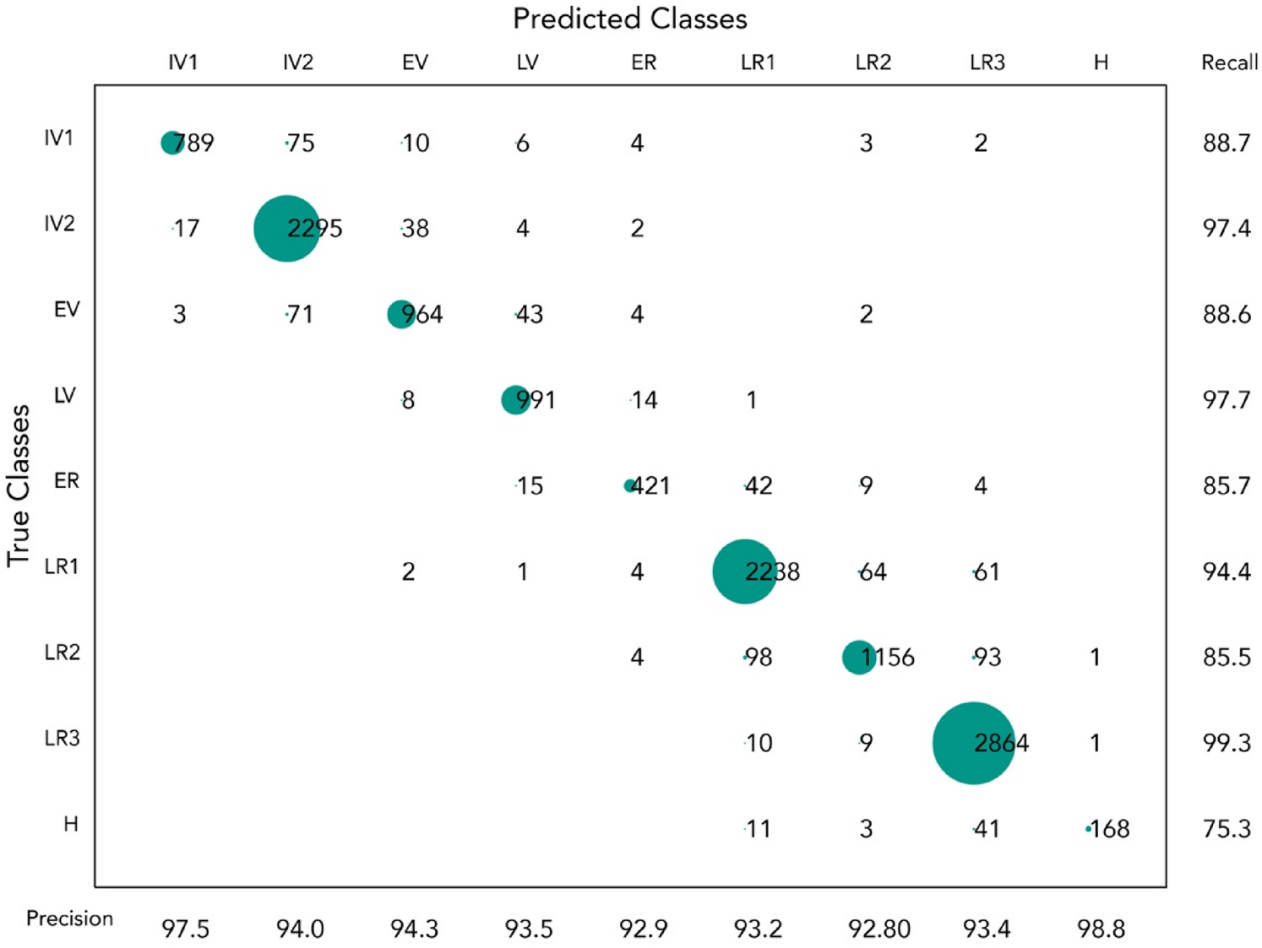
For the years 2013 to 2018, a classification matrix was created that included all classified elements, their magnitude, as well as precision and recall scores. The true classes are on the Y axe, while the model's predictions are on the X axe. The number of fields classified is represented visually by the size of the bubbles; the numbers along the main diagonal represent those successfully classified.

The combination of features optimizing model performance was the following: DOY, EVI, NDVI, NDWI, GCVI, GVMI, VPD, latitude, and longitude. A more detailed view of model performance, spanning all growing seasons from 2013 to 2018 period, is presented as a confusion matrix (Fig. 3). The overall accuracy for this classifier was 94%, (OOB 0.94), and the Kappa coefficient was 0.93. Further details for each year and class are presented in Supplementary Appendix C, Supplementary Table A. Overall, model behavior was similar across classes and years except when the number of elements (supports) was small (< 10). The latter scenario can be visualized with class H for the 2018 year, where only 6 elements were present, and the metrics were considerably smaller compared to other classes with more elements.

#### Classification using VIVPD model across years

Table 2 presents the results when the model was transferred across years. In this analysis the years 2014, 2015, 2017 and 2018 were part of the training dataset, and 2013 and 2016 (dry and wet years respectively) used as validation dataset. The overall accuracy was 93 percent, which was close to the hold-out cross-validation accuracy (Fig. 3). Furthermore, all of the parameters for each class were similar, if not better, with precision values ranging from 0.85 to 1, recall values ranging from 0.85 to 0.99, and f1 scores ranging from 0.86 to 0.97.

**Table 2:** Classification metrics when 2014, 2015, 2017, and 2018 years were used to train the model, and 2013 and 2016 as validation dataset. A total of 14,431 elements (corresponding to the phenological measurements for the years 2013 and 2016) were used for validation and a total of 26,088 elements (corresponding to the p phenological measurements for the years 2013, 2015, 2017 and 2018) were used for training.

Class	f1-score	Precision	Recall
IV1	0.91	0.98	0.85
IV2	0.94	0.94	0.94
EV	0.86	0.85	0.86
LV	0.91	0.85	0.96
ER	0.92	0.94	0.91
LR1	0.96	0.98	0.94
LR2	0.88	0.88	0.88
LR3	0.97	0.95	0.99
H	0.95	1.0	0.90
Accuracy	0.93		
OOB	0.94		

In addition to this split of years, other combinations were tested to observe the behavior when using only wet years for the validation (2015 and 2016) and not present in the training set, and the opposite, allowing only dry years for the validation (2013 and 2018). In both scenarios the overall accuracy was 0.94 for the first approach and 0.95 for the second, and OOB score of 0.95 for both.

#### Classification using VIVPD model across space

The model tested for the spatial transferability of RF resulted on lower values of overall accuracy and low metrics for some classes, such as H, LR2, ER, intermediate for IV1, EV, LR1 and better for IV2, LV and LH3. In all the cases the values were lower when compared against the models previously mentioned (Table 3).

**Table 3:** Classification metrics when testing for spatial transferability using all the years.

Class	f1-score	Precision	Recall
IV1	0.60	0.97	0.43
IV2	0.83	0.77	0.90
EV	0.67	0.73	0.62
LV	0.85	0.80	0.91
ER	0.40	0.60	0.30
LR1	0.65	0.55	0.78
LR2	0.33	0.68	0.22
LR3	0.85	0.76	0.96
H	0.23	0.40	0.15
Accuracy	0.72		
OOB	0.75		

## Discussion

The proposed approach to characterize maize phenology presented the following main advantages: (i) use of publicly available data as model input, (ii) prediction of phenology using high-spatial resolution (30 m), and (iii) use of random forest for classification, allowing the trained model to be easily deployed in GEE for efficient computing processing. These aspects, associated with the stable model performance of phenology prediction when transferred across years, ensures high generalization power. Future uses can be easily adapted for using higher spatial resolution satellite data (e.g., Sentinel 2, 10 m), providing better opportunities for small-scale farming in developing countries.

In exploring a wide feature space for model training and validation (5 weather variables, 5 VIs and 8 surface reflectance bands, latitude, longitude, and DOY for image collection), we found that the full model (considering all 21 predictors) did not perform well. The inferior results for the full model were potentially related to the high correlation among the surface reflectance bands. Predictive models suffer from multicollinearity issues when the independent variables are correlated, causing unpredictable variance in the model outputs (overfitting)^49,50^. In the opposite scenario, a model based solely on geographical features and dates created a potentially spurious ideal classification. When dealing with classification problems, this situation is often avoided by dropping or transforming the features with high cardinality^48^. Even yet, this aspect requires further consideration, particularly when it comes to classification studies integrating agronomical data.

Alternatively, we documented superior performance when VIs and VPD were combined. In addition to VPD^51–53^, VIs NDVI, EVI, and GCVI have been reported as useful in predicting crop development during the growing season, especially for maize and soybean (*Glycine max* L.)^2,17,30^. Furthermore, latitude, longitude, and DOY are strong indicators of how weather and solar radiation patterns influence crop development^54,55^ and dropping them for the rest of the models could result on loosing relevant information.

From a testing standpoint, the model comprised of VI and VPD was stable across all phenology classes, resulting in high classification metrics for the vast majority of them, even in years when weather dynamics substantially impacted crop progress^56,57^ and when tested explicitly for temporal transferability. This latter becomes even more relevant if we consider that the classes correctly classified in all the scenarios correspond with the key developmental stages of the crop, such as emergence, effective density, and yield definition.

These findings align with what was reported by Refs.^45,58^. It is worth mentioning that at least one quality image (with < 30% cloud cover) was retrieved each month between May and September in every year. Although images were available, data retrieved from certain fields was not sufficient to balance the dataset with regard to phenology classes due in part to the characteristics of the ground truth dataset. This resulted in good precision for classes with more observations but poor performance for the underrepresented classes with a smaller number of data points^59,60^. The underperformance of the model transferability across space is also congruent with findings from Refs.^37,45,61,62^. According to Refs.^58,61^ this can be overcome when the territory remains similar as the training dataset, or they share similar weather parameters. Although this could be true for land classification problems, our findings suggest that phenology classification can be more susceptible to these changes.

The lack of quality satellite imagery data, the resulting decrease in the number of classified fields, and the changing environment with the consequent data shift could be remediated with at least three possible solutions not currently addressed by this study. First, thanks to the ability to obtain regular intervals of data for time series analysis, a fusion of satellites^27,63^ would facilitate retrieval of higher-quality data, with improved spatio-temporal resolution preventing data loss, and a better scenario for smoothing techniques such as Savitzky–Golay fitting, locally weighed regression, spline smoothing, and others. Second, because not all classes contained a balanced number of elements, different methods can be applied to managed unbalanced data^64,65^ and ultimately result in a more robust classifier. Lastly, integration of remote sensing data and crop model outputs (i.e., mechanistic- or process-based models) could aid in maximizing predictability power and spatio-temporal limits^66–71^.

## Conclusions

Crop phenology monitoring is crucial for agricultural management since it enables growers, stakeholders, policymakers, and government agencies to determine when the most critical stages are occurring particularly during non-ideal conditions, due to biotic or abiotic stress, where the theory departs from the reality. Is particularly in those cases where models like the one described on this paper can facilitate the report of phenology, and to take more inform decisions. Improving crop phenology classification is becoming more prevalent as current and future research focuses on satellite data fusion and the use of mechanistic or process-based models to enhance spatio-temporal resolution. The findings presented on this paper showed strong classification metrics across years and proven the ability of spectral and weather features to assist in phenology classification. The proposed model also has been tested for spatial transferability and although promising future work should be put on understand the mechanism behind the model behavior.

## Supplementary Information

Supplementary Information.
