# Supplementary material for: An integrated approach of field, weather, and satellite data for monitoring maize phenology
Source: Sci Rep. 2021 Aug 3;11:15711. doi: 10.1038/s41598-021-95253-7 (PMC8333045; doi:10.1038/s41598-021-95253-7)
Supplement: Supplementary file 1 — Supplementary Information. [file 41598_2021_95253_MOESM1_ESM.pdf]

Appendix A Table A.

Summary of variables used for random forest classification models. All variables were extracted from GEE archives with the exception of DOY, latitude, longitude, and growth classes, which were extracted from the ground truth dataset provided by Crop Quest Inc.

Group	Variable name	Description
Bands	B2	Reflectance value for band 2, blue (Landsat 8)
	B3	Reflectance value for band 3, green (Landsat 8)
	B4	Reflectance value for band 4, red (Landsat 8)
	B5	Reflectance value for band 5, near infrared (Landsat 8)
	B6	Reflectance value for band 6, short wave infrared 1 (Landsat 8)
	B7	Reflectance value for band 7, short wave infrared 2 (Landsat 8)
	B10	Reflectance value for band 10, thermal infrared 1 (Landsat 8)
	B11	Reflectance value for band 11, thermal infrared 2 (Landsat 8)
Indices	NDVI	Normalized Difference Vegetation Index
	EVI	Enhanced Vegetation Index

	GCVI	Green Chlorophyll Vegetation Index
	GVMI	Global vegetation Moisture Index
	NDWI	Normalized Difference Water Index
	DOY	Day of the year
	Latitude	Latitude value for the phenology data point
	Longitude	Longitude value for the phenology data point
Weather	GDU	Growing Degree Units
	Precipitation	Cumulative Precipitation
	TMax	Maximum Temperature
	TMin	Minimum Temperature
	VPD	Vapor Pressure Deficit
	Growth Class	Growing Class

Appendix B

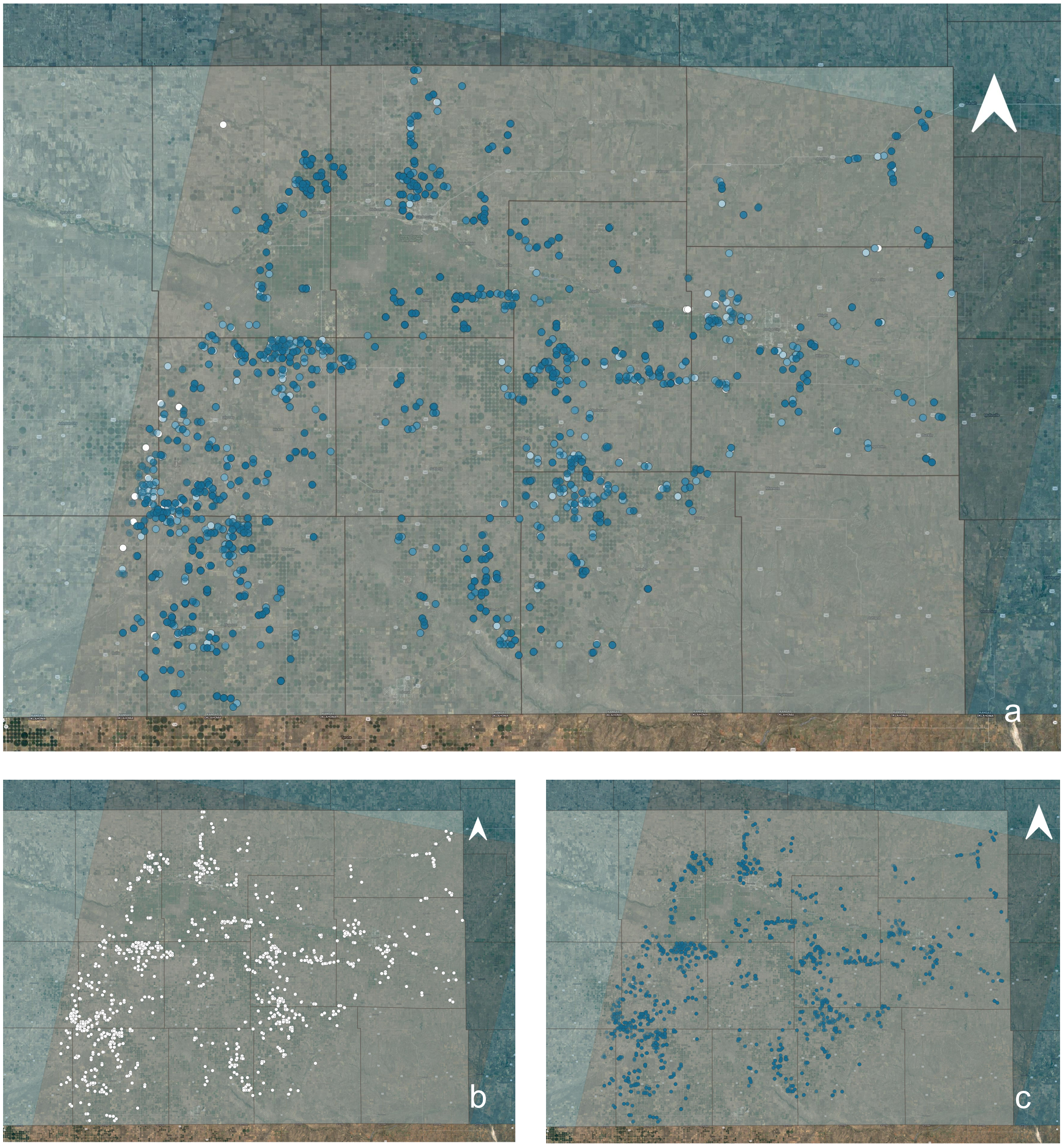

Figure 1: Example of geographical distribution of the phenology samples for 2016. Panel (a) presents both the training and test datasets. Panel (b) white points present the distribution for the test dataset. Panel (c) blue points present the distribution for the training dataset.

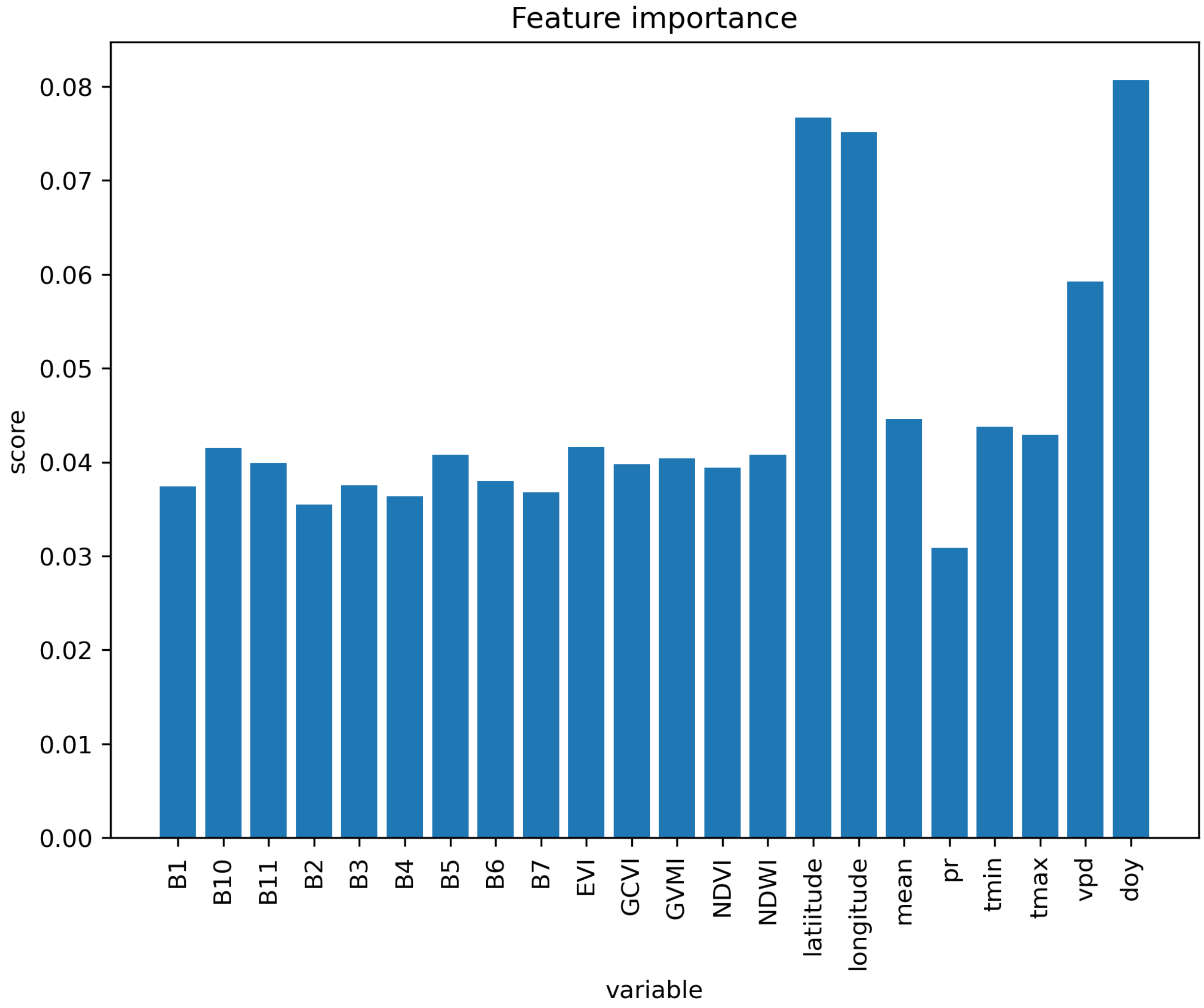

Figure 2: Feature importance analysis for all the variables present in the dataset. The variables are described in Appendix A, Table 1.

Appendix C Table A.

Summary of metrics presenting the results of overall accuracy, OOB, kappa value, precision, recall, F-score, and supports for each class (IV1, IV2, EV, LV, ER, LR1, LR2, LR3, H) from 2013 to 2018. Acronyms defined in Table 1 description.

Year	class	Overall	OOB	Kappa	Precision	Recall	f1-score	test proportion	Training proportion
2013	IV1	0.95	0.95	0.95	1.00	0.96	0.98	195	455
	IV2				0.96	0.98	0.97	344	802
	EV				0.93	0.96	0.94	134	312
	LV				0.99	0.99	0.99	212	494
	ER				0.96	0.88	0.92	50	116
	LR1				0.94	0.95	0.95	389	907
	LR2				0.93	0.87	0.90	264	616
	LR3				0.95	0.99	0.97	546	1274
	H				0.96	0.85	0.90	60	140
2014	IV1	0.97	0.96	0.94	1.00	0.90	0.94	134	312
	IV2				0.98	0.99	0.99	252	588
	EV				0.96	0.97	0.96	111	259
	LV				0.95	0.99	0.97	225	525
	ER				1.00	0.90	0.95	56	130
	LR1				0.93	0.98	0.95	477	1113
	LR2				0.97	0.98	0.93	146	340

	LR3				0.96	0.98	0.97	453	1057
	H				1.00	0.83	0.90	43	100
2015	IV1	0.92	0.92	0.88	0.97	0.84	0.90	189	441
	IV2				0.88	0.96	0.92	331	772
	EV				0.91	0.92	0.91	190	443
	LV				0.99	0.96	0.95	184	429
	ER				0.87	0.98	0.92	77	179
	LR1				0.93	0.90	0.91	296	690
	LR2				0.94	0.85	0.88	322	751
	LR3				0.92	0.98	0.95	501	1169
	H				0.92	1.00	0.96	34	79
2016	IV1	0.96	0.95	0.94	1.00	0.94	0.97	132	308
	IV2				0.97	0.97	0.97	427	996
	EV				0.94	0.95	0.95	264	616
	LV				0.98	0.97	0.98	177	413
	ER				0.98	0.94	0.96	100	223
	LR1				0.93	0.95	0.94	417	973
	LR2				0.91	0.83	0.87	186	434
	LR3				0.96	0.99	0.97	444	1036
	H				0.94	1.00	0.97	17	39
2017	IV1	0.94	0.95	0.93	0.98	0.98	0.98	205	478

	IV2				0.97	0.97	0.97	613	1430
	EV				0.94	0.89	0.92	203	473
	LV				0.95	0.96	0.96	202	471
	ER				0.91	0.96	0.93	121	282
	LR1				0.94	0.98	0.96	477	1113
	LR2				0.96	0.85	0.90	271	632
	LR3				0.95	0.96	0.96	494	1152
	H				1.00	0.97	0.98	61	142
	IV1				1.00	0.82	0.90	91	212
	IV2				0.95	0.99	0.97	439	1024
	EV				0.96	0.88	0.92	161	375
	LV				0.86	1.00	0.93	66	154
2018	ER	0.95	0.94	0.91	1.00	0.86	0.92	42	98
	LR1				0.94	0.94	0.94	267	623
	LR2				0.90	0.88	0.90	159	371
	LR3				0.96	0.98	0.97	410	956
	H				1.00	1.00	1.00	6	14
